# Validation of an In-House High-Throughput Total RNA Sequencing Test for the Detection of Plant Viruses and Viroids

**DOI:** 10.3390/v18060659

**Published:** 2026-06-10

**Authors:** Laëtitia Porcher, Gaël Revert, Léna Créach, Muriel Bahut, Mathieu Rolland

**Affiliations:** 1 Bacteriology Virology GMO Unit, Plant Health Laboratory, French Agency for Food, Environmental and Occupational Health & Safety (ANSES), 7 rue Jean Dixméras, 49044 Angers, France; 2 AgroParisTech, 91120 Palaiseau, France; 3 SFR QUASAV, University Angers, 49000 Angers, France

**Keywords:** HTS, diagnostic, performance criteria, Illumina, ribodepletion

## Abstract

High-throughput sequencing is becoming the method of choice for plant diagnostics. It allows the detection of known and novel viruses and viroids, even in co-infection, without preliminary knowledge of the target. However, this method has its own limitations when compared to real-time PCR or ELISA. Laboratories that implement this type of technologies in-house must ensure that the performance criteria meet the requirements associated with their diagnostic activity. In this study, we present a workflow for in-house plant viruses and viroid detection, based on total RNA extraction, ribodepletion, Illumina sequencing and bioinformatics analyses. Performance criteria such as analytical sensitivity, analytical specificity, selectivity, repeatability, reproducibility and robustness were evaluated on the tomato brown rugose fruit virus (RNA genome), the tomato leaf curl New Delhi virus (DNA genome), and the pepper chat fruit viroid (RNA genome). The performance levels obtained meet the requirements for virus and viroid detection in symptomatic plant samples.

## 1. Introduction

Viruses are ubiquitously present. On plants, they are responsible for important losses and represent a major threat to food security at local, national and global levels [1]. For any pathogen, reliable diagnostic tools have always been key to the management of the associated disease. Since the first description of viruses, scientists have used evolving technologies to detect, characterize, and study these pathogens [2]. After bioassays, electron microscopy, serology and nucleic acid amplification, high-throughput sequencing (HTS) is now the most promising technology for diagnostic development. The real-time PCR has been the gold standard for years now. It allows the design of sensitive, easy to use and reliable detection assays. However, as an a priori method, it requires a preliminary knowledge of the sequence of the target and an assay will have to be used for each target. First used in research, HTS technologies have evolved and are now more widely used. They are increasingly being adopted by diagnostic laboratories. However, these new technologies also have their limitations and laboratories need to demonstrate that the newly developed assays are fit for purpose and meet the requirements associated with their diagnostic activity.

By providing access to the sequence of millions of nucleic acids present in a sample, a HTS test should in theory be able to detect any virus present. Technical choices made by the laboratory at each step of the protocol will, however, impact the performance of the test, and therefore the ability to detect some viruses. In plant pathology, a HTS test can be divided into the main following steps: sampling, nucleic acid extraction, library preparation, sequencing, analysis of raw reads, identification of targets, analysis of controls, target confirmation, interpretation and reporting [3]. Although guidelines exist to help laboratories to design a reliable HTS diagnostic procedure [3,4,5], there is no real consensus and each laboratory designs its own workflow according to its objectives and technical resources (access to the equipment or outsourcing). Some characteristics, such as the depth of sequencing or the completeness of the database used to compare the reads, have an obvious impact on the confidence in the results. The bioinformatic treatment of the data appears to be another critical step [6], and the set-up of a HTS based diagnostic protocol requires bioinformatic skills [7]. This step most often relies on a taxonomic assignment of individual reads or of contigs obtained by de novo assembly by comparison with sequences available in databases [3]. However, tools based on deep learning strategies have recently been developed and trained to facilitate the discovery of novel viruses [8,9].

Before using a diagnostic test, a laboratory should characterize the assay in order to identify its limitations. In plant pathology, guidelines are provided concerning the different performance criteria to evaluate and the methodology to follow [10]. The main criteria correspond to the analytical sensitivity, analytical specificity (inclusivity and exclusivity), selectivity, repeatability, reproducibility and robustness. However, these guidelines were developed with earlier technologies in mind and HTS is a technology for which it is still too costly to achieve the number of samples recommended for validation. Several validations of HTS routine tests have already been described in various contexts [11,12,13,14].

A workflow has been set-up for in-house official control and detection of emerging and/or novel viruses. It is based on total RNA extraction, ribodepletion and Illumina sequencing. By targeting total RNA, the protocol allows the detection of RNA viruses, DNA viruses and viroids [15]. For routine use, ribodepleted total RNA and sRNA sequencing are easy to implement. However, for virus detection, ribodepleted RNA sequencing seems more appropriate [15]. Sequencing is performed in-house on the most affordable Illumina systems (iSeq or MiSeq). The bioinformatic analyses are based on a previously developed pipeline [16], modified to implement a taxonomic assignment by protein sequence alignment [17] and a deep learning tool for novel viruses detection [8]. The present publication describes the validation process of the workflow intended to be used for the detection of known and unknown RNA viruses, DNA viruses and viroids. The validation was performed on tomato brown rugose fruit virus (ToBRFV, Tobamovirus, RNA genome), tomato leaf curl New Delhi virus (ToLCNDV, Begomovirus, DNA genome) and pepper chat fruit viroid (PCFVd, viroid, RNA genome).

## 2. Materials and Methods

### 2.1. Biological Material

Most of the samples selected for this study (Table 1) have been collected in the framework of official controls. Sample NPPO-NL 4972392 has been provided by NIVIP [18]. Most were collected fresh and stored at −80 °C, some were freeze-dried. These samples did not necessarily present symptoms but have previously been characterized by PCR or sequencing.

### 2.2. Sample Preparation

Individual samples were weighed in Bioreba bags and stored at −80 °C. The sample size was 1 g for fresh plant material and up to 0.3 g for freeze-dried plant material. Plant material was ground in 4.5 mL of GI lysis buffer (4 M guanidine isothiocyanate, 0.2 M sodium acetate pH 5.2, 25 mM EDTA, and 2.5% PVP-40) using a ball mill (Homex 6, Bioreba, Reinach, Switzerland). A total of 450 μL of the obtained ground material was transferred into a microtube with 45 μL of Sarkosyl (=N-Lauroylsarcosine) and incubated at 70 °C (1500 rpm, 10 min).

A mixture of three contaminated samples stored at −80 °C (Table 2) was prepared as follows: 1 g of zucchini leaves infected with ToLCNDV and 0.5 g of tomato leaves infected with PCFVd were ground in 4.5 mL of GI lysis buffer. Then, 100 μL of ground leaf from tomato infected with ToBRFV were added. Considering our standardized preparation protocol of samples for routine testing (1 g of plant material ground in 4.5 mL of buffer), we considered that there was no dilution of ToLCNDV and PCFVd and that the ToBRFV infected matrix was diluted 45 times.

### 2.3. Evaluation of the Performance Criteria

**Figure 1:**
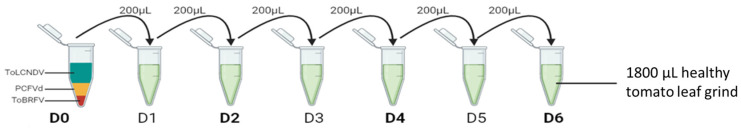
Dilution range of the mixture containing the viruses and viroid studied. Dilutions of 1/10 were carried out in ground material from healthy tomato leaves.

To test the analytical sensitivity, one serial dilution of the mixture was prepared in ground healthy tomato leaves (sample GH2). The dilutions 10^−2^ (D2), 10^−4^ (D4) and 10^−6^ (D6) were prepared (Figure 1). The undiluted mixture (D0) and dilutions (D2, D4 and D6) were used in 3 independent replicates (extraction, library preparation, sequencing).

To test the analytical specificity, 11 plant samples infected with Tobamovirus, Begomovirus and viroids were analyzed (Table 1).

To test the selectivity, grinds from plants from which RNA extraction is considered difficult (*Vitis* sp. and *Euphorbia* sp.) were spiked with the mixture. Exactly 100 μL of the mixture were added to 350 μL of ground material from the *Vitis* sp. and *Euphorbia* sp. leaves in order to obtain the 450 μL necessary for the extraction.

To test the reproducibility, libraries were prepared in parallel by two operators from a blind sample previously characterized by a different laboratory and showing low RIN (sample NPPO-NL 4972392 [18]). Sequencing runs of the three libraries obtained from the mixture diluted at 10^−2^ were also performed on two different Illumina sequencers, the MiSeq and the iSeq 100 (Illumina, San Diego, CA, USA).

### 2.4. Total RNA Extraction

Total RNA was extracted using the commercial RNA Plant Mini Kit (QIAGEN, Hilden, Germany) according to the supplier’s procedure “Purification of Total RNA from Plant Cells and Tissues and Filamentous Fungi”. During the procedure, samples were treated on-column with DNase (QIAGEN, 80 μL per sample) to degrade the plant genomic DNA. Total RNA was eluted in 50 μL of molecular grade water and stored at −80 °C.

During each RNA extraction, a negative isolation control (NIC) was included, *Nicotiana tabaccum* xanthi leaves which were processed as a regular sample. This control sample is routinely included in the library preparation.

Quantifications and quality controls of extracted total RNAs were performed using, first the High Sensitivity Qubit RNA kit (ThermoFisher Scientific, Waltham, MA, USA) with a Qubit 2.0 fluorometer (Invitrogen, Darmstadt, Germany), then the RNA 6000 Nano kit on a Bioanalyzer 2100 (Agilent Genomics, Santa Clara, CA, USA). Assays were performed on 1 μL of extract according to the supplier’s protocols.

### 2.5. Library Preparation

**Figure 2:**
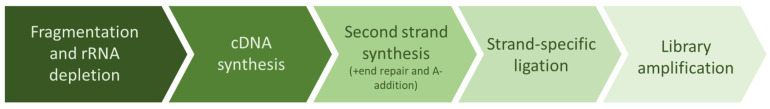
Different steps of the library construction protocol for the total RNA sequence of plant samples, according to the protocol of the QIAseq Stranded RNA Library Enzyme kit (QIAGEN).

The protocol used was based on the QIASeq Stranded RNA Library Enzyme kit (QIAGEN). The library preparation consisted of several steps (Figure 2). During fragmentation, the QIAseq FastSelect rRNA Plant kit (QIAGEN) was used to ensure the depletion of ribosomal RNA (rRNA) by specific binding of oligonucleotides, preventing their reverse transcription into cDNA.

For library preparation, RNA extracts were normalized to 800 ng in 23 μL, in microtube strips. During this normalization, 2 μL of ERCC RNA Spike-In Mix (External RNA Controls Consortium, Invitrogen) was added to the negative control NIC.

Total RNAs were enzymatically fragmented and then ribodepleted with the QIAseq FastSelect rRNA Plant kit. The duration of the fragmentation step depended on the integrity of the extracts (RIN): 15 min for good integrity RNA (RIN > 8), 10 min for average integrity RNA (RIN 5–8), 5 min for degraded RNA (RIN 3–4) and no fragmentation for poor integrity RNA (RIN < 3).

After the fragmentation/ribodepletion step, the preparation of the libraries was carried out according to the supplier’s recommendations with the following adaptations. In this study, the unique dual indices adapters were diluted 1/25. The cleaned libraries were amplified during 10 PCR cycles, and the amplified libraries were purified twice with a ratio beads/DNA of 0.9 (*v*/*v*) to ensure the proper elimination of adapter dimers.

Purified DNA libraries were quantified on 1 μL using the High Sensitivity Qubit DNA kit (ThermoFischer) on a Qubit 2.0 fluorometer (Invitrogen). To verify the absence of adapter dimers and to determine the size of the libraries, quality controls were performed using the High Sensitivity DNA kit on the Bioanalyzer 2100 (Agilent Genomics). To be sequenced, a library had to present a distribution of DNA fragments sized between 300 and 500 bp and a concentration of dimers representing less than 2% of the total library yield.

### 2.6. Sequencing

For MiSeq sequencing, 13 libraries (including the healthy control (NIC) containing the ERCC) were pooled equimolarly. The quantification of the pool was checked by qPCR using the KAPA library quantification kit for Illumina platforms (Roche, Basel, Switzerland).

For iSeq100 sequencing, 4 libraries (including the healthy control (NIC) containing the ERCC) were pooled and checked as previously described.

PhiX Control v3 (FC-110-3001, Illumina, San Diego, CA, USA) was added to the pools at 5% and loaded at 12.5 pM (MiSeq) or 125 pM (iSeq100) on cartridges of Reagent v2 300 cycles kit (Illumina) following the supplier’s instructions. The sequencing run conditions were 2 × 149 bp and dual indices of 10 bp each.

### 2.7. Bioinformatic Analysis

The bioinformatic analyses were performed using a previously developed pipeline [16] optimized for taxonomic assignment and discovery of novel viruses.

The paired-end sequences were bioinformatically analyzed on the Migale cluster farm (https://migale.inrae.fr, accessed on 15 April 2026). Unless specified otherwise, default parameters were used. The raw reads were trimmed and filtered according to the PHRED score using Fastp v0.24.0 [19]: bases from the 3′ end were trimmed if the mean quality score in a 3 bases sliding window was below 20. Reads shorter than 36 nucleotides were deleted. A de novo assembly of the cleaned reads was performed with Spades v4.1.0 [20], using—rnaviral option.

The obtained contigs were then subjected to viral sequence detection using the VirHunter generalistic model [8], as trained by its authors and to taxonomic assignment with Diamond v2.1.11 [17] on the NCBI non-redundant protein collection (September 2025) using the more-sensitive option. Viruses for which at least one contig of 250 bp or more, obtained from at least 10 reads and considered viral by VirHunter and Diamond, showed an identity above 65% and an e-value below 0.01 were suspected to be present. A list of keywords (see Table S1) was used to filter the list of suspected viruses, eliminate animal viruses, phages and mycoviruses and limit further analysis to potentially present plant viruses.

Contigs considered viral by VirHunter but not assigned to viral sequences by Diamond were considered separately for the potential detection of novel viruses.

The non-assigned contigs were compared, using BlastN v2.16.0 [21], to a local database containing viroids sequences. This database was obtained by downloading sequences labeled as “viroid” [Title] and “viruses” [Organism] from the NCBI non-redundant nucleotide collection. Viroids for which at least one contig showed an identity above 65% and an e-value below 0.01 were suspected to be present.

The presence or absence of ToBRFV, ToLCNDV, PCFVd was confirmed by mapping the trimmed reads to reference genomes (1 for ToBRFV (QBANK23289), 2 for ToLCNDV (DNA-A: QBANK22939, DNA-B: QBANK22940; DNA-A: QBANK24440, DNA-B: QBANK24441), 1 for PCFVd (QBANK22265)) downloaded from the EPPO-Q-bank (https://qbank.eppo.int, accessed on 15 April 2026) with Bowtie 2 v2.5.4 [22] and Samtools v1.21 [23]. For other suspected plant viruses and viroids, the reads were mapped to the nucleotide sequence corresponding to the best Diamond or Blast hit of the longest contig, which was downloaded from NCBI.

Suspected viruses and viroids were considered absent if reads covered less than a third of the reference sequence. Above a third of total coverage, an indicator corresponding to the consecutive covered bases (CCB; percentage of the reference sequence represented by the longest region where the number of consecutive bases are covered) was used. For a CCB above a third of the reference sequence size, the virus, viroid or a close relative was considered present. For other cases, it was inappropriate to have an automatic assignment of results; an expertise was required based on all available results such as, the mean read depth (MRD, sum of the mapped read depths at each reference base position, divided by the number of known bases in the reference), the possible attribution of the coverage to a different virus or the probability to find the virus in the sequenced plant material. In some cases, confirmation by a different method might be required. The use of expertise of operators is especially valuable for newly detected viruses usually providing good coverage but a poor similarity level. In such cases, contigs and/or consensus sequences were compared with the nt database (NCBI), ICTV criteria were used to determine if the sequence should be attributed to a new species.

If a virus or viroid was detected in the NIC, the experiment would have to be repeated. If a high number of virus or viroid reads were found in the NIC, while remaining negative, this should be taken into account during the exploitation of results for samples requiring the expertise of the operator. Detected ERCC fragments were identified by mapping the reads to sequences of the ERCC fragments. For each run, the percentage of ERCC fragments found at least once in the negative control was calculated. This percentage was monitored between sequencing runs to ensure the stability of the results. The mean value and standard deviation of this indicator were calculated considering the results of all the sequencing runs performed during this validation study. For each run, the value of this indicator had to exceed the mean value minus three times the standard deviation (i.e., 35).

### 2.8. Confirmation by PCR and Sanger Sequencing

When possible, the presence of viruses, viroids or satellites considered present was confirmed by PCR using the primers, probes and kits listed in Table 3, followed by Sanger sequencing for non-specific reactions. After cleaning, obtained sequences were compared, using BlastN [21], to the NCBI non-redundant nucleotide collection.

## 3. Results

Most experiments were performed using a mixture of samples infected by ToBRFV, ToLCNDV or PCFVd (as described in materials and methods). Before preparing the mixture, each sample was sequenced separately to ensure that the other two targeted viruses/viroid were not present in the sample. In order to ensure the appropriate representation of each virus or viroid, the obtained results were also used to determine the relative ratio of each sample in the final mixture. All data associated with the different sequencing runs and the results of the mappings to reference genomes of ToBRFV, ToLCNDV or PCFVd (MRD, coverage and CCB) are presented in Table S2 in Supplementary Material. The taxonomic assignment and mapping results obtained for the samples used to characterize the specificity are provided respectively in Tables S3 and S4 in Supplementary Material.

### 3.1. Sequencing Data Quality

The number of reads obtained for each sample and an indicator of the quality of the reads are shown in Table S2. The number of reads obtained ranged from 1.3 to 4.9 million, with a median value of 2.4 million. After filtering, the percentage of bases with Q30 and above ranged from 94.5 to 98.9.

### 3.2. Controls Analysis

The number of ERCC fragments found at least once in each negative control is provided in Table S2. During the validation study, the mean value and standard deviation of this indicator were respectively 43.2 and 2.68. For all the sequencing runs, the number of ERCC fragments found at least once was above the standard deviation minus three times the standard deviation (i.e., 35).

Negative controls were analyzed in the same way as the samples to detect any potential cross contaminations during sample preparations, from extraction to sequencing. The results of the detection for each control (from GH1-1 to GH1-5) are reported in Table S2. The coverage values observed in the negative controls were below the positivity threshold, the highest value was an 8.1% coverage of the ToBRFV genome in GH1-4. No other viruses of viroids were detected in the negative controls.

### 3.3. Status of Samples Used to Prepare the Mixture

Sequencing samples 20/13,4 22/4243 and 12/456i22 confirmed the presence of ToBRFV, ToLCNDV and PCFVd, respectively. Results show a difference in MRD of almost a factor 150 between ToBRFV (MRD above 38,000 in sample 20/13,4) and ToLCNDV (MRD of 260 on DNA-A in sample 22/4243). Unexpected viruses were also detected in sample 22/4243 (watermelon mosaic virus, Moroccan watermelon mosaic virus and cucurbit aphid-borne yellows virus) and 12/456i22 (tobacco vein clearing virus), however their presence was not considered problematic for the ongoing validation.

### 3.4. Analytical Sensitivity

The analytical sensitivity of the method for ToBRFV, ToLCNDV and PCFVd was determined using the sample mixture in four different concentrations: not diluted, 10^−2^, 10^−4^ and 10^−6^ dilutions.

The detection of each virus/viroid for the three technical replicates is reported in Table 4. The final dilution of the virus or viroid takes into account the dilution of the virus in the mixture, and the dilution factor of the mixture.

Among the three viruses or viroids in the mixture, ToBRFV is the virus for which the method presented the best sensitivity. The virus was detected in the three replicates in the 10^−2^ diluted mixture which, with the original dilution of the sample in the mixture, represented a dilution of the original sample of 2.2 × 10^−4^.

Using the same dilution of the mixture, corresponding to a final dilution of 10^−2^, the described method could detect both ToLCNDV and PCFVd reproducibly. However, expertise was required to assign a positive result to one replicate of ToLCNDV (coverage of 89.7% and CCB of 24.4%).

### 3.5. Selectivity

**Figure 3:**
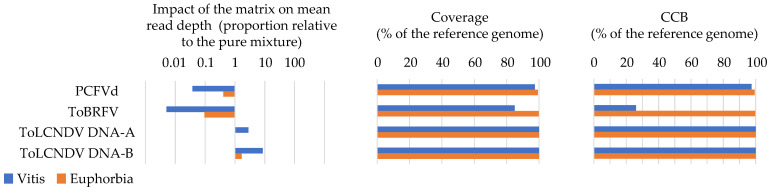
Evaluation of the selectivity of the method through the impact of the matrix on mean read depth (reads, on average, likely to be aligned at a given reference base position), coverage (percentage of the reference sequence covered) and CCB (percentage of the reference sequence represented by the longest region where the number of consecutive bases are covered), obtained for PCFVd, ToBRFV and ToLCNDV in samples mixed with *Vitis* sp. and *Euphorbia* sp. tissues.

To test the selectivity of the method, the sample mixture was mixed with ground material of matrices for which the RNA extraction is considered difficult: *Vitis* sp. and *Euphorbia* sp. leaves. The obtained extracts respectively had concentrations of 228 and 198 ng/µL and RIN values of 5.9 and 6.1. Their RIN were above the average value obtained during the validation. After filtering, the total number of reads obtained was 1.7 for *Vitis* sp. and 3.3 million for *Euphorbia* sp. The MRD, coverage and CCB results obtained are shown in Figure 3.

Compared to the results obtained for the pure mixture, the mean read depth for PCFVd and ToBRFV decreased in *Vitis* sp. by factors of more than 10 and 100 respectively, while an increase was observed for ToLCNDV. Similar tendencies were observed to a lower extent in *Euphorbia* sp. On both matrices, coverage remained above 80%; only the CCB observed for ToBRFV in *Vitis* sp. felt below the automatic assignment threshold.

### 3.6. Analytical Specificity

The analytical specificity of the method was evaluated based on the results of the three samples containing the selected targets (PCFVd, ToBRFV and ToLCNDV), one unknown positive sample, and seven samples containing none of the selected targets but one of their close relatives (Table 5 and Table S4).

Two samples, each containing a tobamovirus closely related to ToBRFV (tomato mosaic virus and tobacco mild green mosaic virus), were tested. The correct virus was identified and the results for ToBRFV were negative in each sample.

The results are equivalent for ToLCNDV and PCFVd: in the samples with a close Begomovirus or viroid, the method allowed the detection of the present virus(es) and the result was negative for ToLCNDV and PCFVd. For samples EL 20 30031 and 17/104,1, different Begomoviruses were selected for mapping, all of which provided a coverage and a CCB above the 33% threshold. For sample EL 20 30031, the sequence of tomato geminivirus Senegal used for mapping is a partial sequence showing 98.3% identity with the full genome of tomato leaf curl Mali virus also included in the mapping. For sample 17/104,1, three contigs corresponded to Begomovirus sequences (Table S3). Further analysis showed that nodes 59 and 61 respectively corresponded to the chili leaf curl virus and pepper leaf curl, node 2672 corresponded to both viral species.

Where possible, the results obtained using the HTS method were confirmed by PCR and Sanger sequencing (Table S4). All the results obtained conformed to expectations. All PCR targeting, specifically or not, a virus or viroid detected using HTS were positive. When the Sanger sequencing provided interpretable results, Blast assignment consistently allowed the identification of the virus or viroid detected using HTS method and targeted by PCR (or of one of these when PCR enabled the amplification of several viruses).

### 3.7. Repeatability and Reproducibility

Repeatability was assessed with the three replicates of each dilution tested during the analytical sensitivity analysis (Table 4 and Table S2). When taking into consideration the automatically assigned qualitative results, the method showed a 94.4% level of repeatability. The relative standard deviation between repeats at concentrations allowing the detection of the target ranged from 8% to 69% for the MRD, from 0% to 28% for the coverage and from 0% to 80% for the CCB.

**Figure 4:**
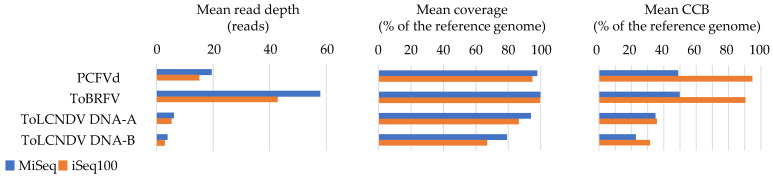
Evaluation of the reproducibility of the method through the mean read depth (reads, on average, likely to be aligned at a given reference base position), coverage (percentage of the reference sequence covered) and CCB (percentage of the reference sequence represented by the longest region where the number of consecutive bases are covered), obtained for PCFVd, ToBRFV and ToLCNDV for samples D2 processed by different operators and sequenced on MiSeq or iSeq100.

The reproducibility was evaluated by testing sample NPPO-NL 4972392 while modifying the operator and by testing the three libraries obtained from sample D2 while modifying the sequencer. For sample NPPO-NL 4972392, the two libraries and sequencing runs allowed the detection of the same virus with a MRD corresponding to the same proportion of the total number of reads obtained for the sample. In each case, coverage and CCB were close to 100%. The results obtained for D2 replicates are shown in Figure 4.

ToBRFV and PCFVd were automatically detected in the three repeats, while ToLCNDV was automatically detected in only one. The results obtained for MRD and coverage were comparable between the two experiments, with a ratio between values ranging from 0.75 to 1. Higher CCB values were obtained for ToBRFV and PCFVd during the experiment conducted on iSeq100, with ratios of 1.8 and 1.9.

## 4. Discussion

High-throughput sequencing has enabled the detection and identification of numerous new viruses [35], making it the new gold standard for molecular detection methods without a priori. However, the use made of the technology will highly impact the performance level of the method. This performance will not only depend on the proficiency of the laboratory, but also on the resources allocated to implement the method (i.e., sequencing depth, computational resources). Each step of the presented assay has been designed to optimize the chances of detecting viruses and viroids present in all types of plant matrices, regardless of the composition (DNA or RNA) or size of their genome, while remaining usable in-house as a routine tool.

During the development process, the choice of sequencing total ribodepleted RNA with Illumina over other strategies, such as sRNA or dsRNA sequencing, or other sequencing platforms (ONT, Pacbio) was based on the literature [15], but also on the laboratory’s experience. Ribodepletion allows up to tenfold enrichment of viral RNAs [36]. In addition, the use of a kit that blocks the reverse transcription of ribosomal RNA into cDNA (as used in our protocol), limits the loss of nucleic acid during bead capture. Results obtained during the validation process showed sufficient quality levels.

The developed assay is intended to be used by a laboratory under quality assurance process and required the appropriate controls. A single healthy sample spiked with RNAs during the preparation of the library was used to cover the different needs. The RNA spike was composed of a common set of RNA controls usable for qPCR and HTS. It is composed of 92 RNAs, plasmid DNA transcripts, of known sizes and concentrations. The proportion of these synthetic RNA found among the reads obtained for the control is monitored. Based on the results of this study, a threshold has been set to ensure the stability of the performance characteristics between runs. In our experimental conditions, 35 fragments out of 92 should be detected at least once. An indicator below this threshold should warn the operator of a problem during one step of the analysis or the use of a degraded ERCC. Contamination was also verified by confirming the absence of viruses in the controls. During the experiments, very few viral reads were found in the controls, with a coverage below one-fourth of the threshold set to consider the presence of a virus (33%).

During the validation process, two viruses (ToBRFV, RNA genome; ToLCNDV, DNA genome) and one viroid (PCFVd) of interest were selected to demonstrate the ability of the method to reliably detect the different targets for which the assay was developed. This work aimed to characterize the performance level of the entire analytical process. To reflect the processing of real independent samples, the results were not normalized based on a certain number of reads before being processed and compared.

Sample preparation followed the laboratory’s standard diagnostic procedure (weighing and grinding in plastic bags). The use of Sarkosyl (a detergent used in concentrated saline solutions to solubilize cellular components) and guanidine isothiocyanate buffers made the assay suitable even for difficult matrices. The selectivity of the assay was tested on *Vitis* sp., which is rich in polyphenols that can precipitate with RNA and interfere with the analysis [37], but also on *Euphorbia* sp., which contains latex composed of polysaccharides [38]. Extracting RNA from these matrices was difficult because the samples became viscous during grinding. The obtained RIN values were average and very similar for the two tested samples. However, the number of reads obtained from the *Vitis* sp. sample was only half that obtained from the *Euphorbia* sp. sample. In *Vitis* sp. the MRD for PCFVd and ToBRFV was highly impacted, with a decrease by factors of more than 10 and 100, respectively. The coverage and CCB for PCFVd remained above 97%, allowing the automatic assignment of a positive result. For ToBRFV, coverage was 85%, but CCB was more impacted and fell to 26%, requiring the expertise of the operator to conclude to a positive result. Results obtained for the tested DNA virus (ToLCNDV) showed little impact of the tested matrices. This experiment allowed us to highlight the possible impact of the matrix. The detection of the viruses and viroids tested remained, nevertheless, successful on spiked samples.

For the two sequencers used, the results presented were obtained from cartridges designed to provide raw sequences (reads) of 150 bp in both directions (up to 15 million reads per direction on the MiSeq and up to 4 million reads per direction on the iSeq 100 cartridge). Cartridges were used to simultaneously sequence 13 samples on the MiSeq and four samples on the iSeq100, with approximately 2 million reads expected for each sample (including the control sequenced during each run). Results showed a high disparity in the number of reads obtained per sample (up to a 4-fold ratio), despite the equimolar pooling of the libraries. The problem could be due to a difficulty in determining the size of the libraries, or a difference in quality. It would be intuitive to think that sensitivity is highly dependent on the depth allocated to sequencing: the more sequences obtained for each sample, the more likely the assay is to detect a pathogen present at a low level in the sample. However, preliminary tests and bioinformatic sub-sampling of reads led us to conclude that even with less than 1 million reads the results obtained were suitable for the detection of the three used targets and, more globally, for diagnosis of symptomatic plant samples. Previous studies have also shown that sensitivity depends more on genome size, virus type and background nucleic acids amount [39].

In plant virology, the analytical sensitivity (smallest amount of target that can be detected reliably) is determined by testing serial dilutions of an infected sample tissue in healthy plant tissue [10]. In the present study, the assay demonstrated the ability to detect ToBRFV at a dilution as low as a 10^−4^ (10^−2^ for ToLCNDV and PCFVd) which is appropriate considering the scope of the method (diagnostic on symptomatic plant tissues). The analytical sensitivity has been confirmed on iSeq100, and is comparable to that previously described for this technology with other viruses [11]. For ToLCNDV, the dilution of 10^−2^ was probably very close to the detection limit of the method. At this dilution, a limited repeatability was observed in the automatically assigned results. Determining the analytical sensitivity from naturally infected and uncalibrated material, as well as the analysis of non-normalized data, reflect the viral load present in natural samples such as those that will be tested in diagnostics. Nevertheless, it makes it difficult to compare results from one virus to another. The highest sensitivity observed for ToBRFV is indeed most likely explained by the high titer of this virus in plants (MRD above 38,000 in sample 20/13,4). This result could also be due to the choice of nucleic acid type for the library preparation: a better recovery of specific reads from ssDNA viruses or viroids was observed when sequencing sRNA, whereas rRNA depleted total RNA tended to foster RNA linear genomes [15]. Several studies have described the ability of HTS to detect viruses as comparable to RT-PCR [40] or hybridization [41]. However, the strength of this technology lies in its ability to detect unexpected or even unknown viruses or viroids.

When limited to the detection of two viruses and one viroid, bioinformatic analyses are straightforward. Nonetheless, the assay was designed to be able to detect any virus of viroid. The inclusivity of the method mainly relies on the used database (NCBI). However, the use of a tool based on deep learning to filter out the non-viral sequences represents a risk of missing sequences of interest. VirHunter, the deep learning convolutional neural network (CNN) used here, was trained in 2022 on sequences found in the NCBI to differentiate any viral sequence from plant and bacterial sequences [8]. The CNN provided satisfactory results; by filtering out non-viral sequences, it facilitated the analysis of the results and enabled the automation of mapping. In all the analyzed samples, the discrimination provided by the CNN, while not always accurate, allowed the detection of the expected viruses and viroids as well as the detection of unexpected viruses or viroids.

By detecting the three selected targets and some of their close relatives, the designed assay demonstrated the expected level of exclusivity. To conclude on the presence or absence of a virus or viroid, the trimmed reads are mapped to a reference genome or sequence. Considering the low intraspecific diversity of ToBRFV and PCFVd, only one genome was used, whereas two were used for ToLCNDV. For other species, closer attention should be given to the results of mappings performed on several closely related viruses or viroids to determine whether the sample is co-infected or whether the mappings simply reflect the genetic identity of the sequences used for mapping. For example, in sample 17/104,1 mappings performed on five different sequences corresponding to full genomes of five different Begomoviruses reflected the presence of two different species in the sample. This situation was also facilitated by the very high genetic identity between Begomoviruses species.

There is often a trade-off between method performance and available resources. A trade-off also exists concerning the validation process. For cost reasons, the presented validation study did not comply with all the recommendations developed for the validation of diagnostic assays based on previous technologies. Multiplying the samples to demonstrate the inclusivity and exclusivity of the method required too many resources for a limited increase in confidence considering the scope of the method.

Despite guidelines for the implementation of HTS tests in plant diagnostics laboratories, defining criteria for the interpretation of the controls and samples remains a difficult task in the development of a new assay and some experience is required before reliable thresholds can be established. Thresholds are decision support tools meant to facilitate the assignment of a result. One drawback of this approach is that it can limit the ability of the method to detect viruses or viroids at low concentrations. However, to limit this risk, established thresholds are based on coverage rather than on read depth. Furthermore, their use also ensures the accuracy of the results and reduces the risk of false assignment. From this perspective, CCB is especially valuable to distinguish a virus from a close relative (known or unknown). For sample 19/70, which was contaminated by tomato mosaic virus, mapping the reads to the reference genome of ToBRFV showed 50.6% coverage, but only 9.56% CCB, enabling the operator to clearly attribute the results to the presence of tomato mosaic virus. As previously described, the high level of genetic identity between the Begomovirus species present in sample 17/104,1 did not allow their automatic identification and further analysis of the results was required. Considering the amount of data provided by the analysis and the different biases, the expertise is still required, especially in cases approaching the thresholds. In some cases, the confirmation of a result by PCR or any other appropriate method might be needed. Thresholds set up by the authors simply reflect their experience. In their experimental conditions, these thresholds provided the expected level of quality.

According to the results obtained, the test is considered fit for purpose and will be used on samples for which regular diagnostic tools are unable to detect a pathogen, while the symptomatology or the microscopic observation suggests a virological origin of the symptoms observed.

## Figures and Tables

**Table 1:** Samples selected for the study to evaluate the main performance criteria (sensitivity (Sens.), specificity (Spe.), selectivity (Sel.), repeatability (Repeat.) and reproducibility (Repro.)). The status of healthy samples was confirmed by HTS. Freeze-dried samples are symbolized with *.

Sample	Plant Species	Virus or Viroid Present	Viral Genus	Criteria Evaluated				
				Sens.	Spe.	Sel.	Repeat.	Repro.
20/13,4	*Solanum* *lycopersicum*	tomato brown rugose fruit virus (ToBRFV)	Tobamovirus	X	X	X	X	X
22/4243	*Cucurbita pepo*	tomato leaf curl New Delhi virus (ToLCNDV)	Begomovirus	X	X	X	X	X
12/456 i22	*Solanum* *lycopersicum*	pepper chat fruit viroid (PCFVd)	Pospiviroid	X	X	X	X	X
NPPO-NL 4972392 *	*Oxalis* *triangularis*	shamrock chlorotic ringspot virus (SRSV)	Potyvirus		X			X
08/01,1 i17 *	*Nicotiana* *benthamiana*	tomato chlorotic dwarf viroid (TCDVd)	Pospiviroid		X			
16/324,3 *	*Dahlia* sp.	dahlia latent viroid (DLVd)	Hostuviroid		X			
19/70	*Solanum* *lycopersicum*	tomato mosaic virus (ToMV)	Tobamovirus		X			
23/1341	*Solanum* *melongena*	tobacco mild green mosaic virus (ToMGMV)	Tobamovirus		X			
EL 20 30031 *	*Solanum* *lycopersicum*	tomato leaf curl Mali virus (ToLCMLV)	Begomovirus		X			
EL 25 12/49 *	*Solanum* *lycopersicum*	tomato leaf curl Comores virus (ToLCV)	Begomovirus		X			
17/104,1 *	*Capsicum* *annuum*	chili leaf curl virus (ChiLCV)	Begomovirus		X			
21/202	*Vitis* sp.	healthy plant				X		
21/4563	*Euphorbia* sp.	healthy plant				X		
GH1	*Nicotiana tabacum* xanthi	healthy plant						
GH2	*Solanum* *lycopersicum*	healthy plant						

**Table 2:** Details of the prepared viruses and viroid mixture.

Sample	Plant Species in the Mixture	Target Virus or Viroid	Contribution in the Mixture
20/13,4	*Solanum lycopersicum*	tomato brown rugose fruit virus (ToBRFV)	dilution 1/45
22/4243	*Cucurbita pepo*	tomato leaf curl New Delhi virus (ToLCNDV)	no dilution
12/456 i22	*Solanum lycopersicum*	pepper chat fruit viroid (PCFVd)	no dilution

**Table 3:** Primers and PCR kits (Platinum Taq DNA Polymerase (Invitrogen) or Ag-Path-ID one-step RT-PCR (Thermo Fisher Scientific)) used for result confirmation. Platinum.

Target	Primers (Probes)	PCR Kit
Begomovirus	Beg CP-F/Beg 580R [24]	Platinum
	TY1/TY2 [25]	Platinum
Betasatellite	CLB 36F/CLB 37R [26]	Platinum
Caulimovirus	C-cpF/C4281 [27]	Platinum
Ilarvirus	Ilar2F5/Ilar2R9 [28]	Ag-Path-ID
Polerovirus	PolGenUp2/PolGenDown2 [29]	Ag-Path-ID
Pospiviroid	Pospi1 RE/Pospi1 FW [30]	Ag-Path-ID
Potyvirus	CiFor/CiREV [31]	Ag-Path-ID
STV	STV-F/STV-R [32]	Ag-Path-ID
Tobamovirus	TobamodF/TobamodR [33]	Ag-Path-ID
ToBRFV	ToBRFVqs1/ToBRFVqas2 (ToBRFVp1) [34]	Ag-Path-ID

**Table 4:** Detection of the three viruses and viroids in the mixture according to the dilution. The final dilution takes into account the dilution of the virus in the mixture. For each technical replicate, the detection of the virus is positive or negative. In case of non-automatic assignment (assignment based on the expertise of the operator, see Section 2.7), the result is indicated between brackets.

Mixture Dilution	ToBRFV		ToLCNDV		PCFVd	
	Final Dilution	Detection (3 Replicates)	Final Dilution	Detection (3 Replicates)	Final Dilution	Detection (3 Replicates)
Not diluted	2.2 × 10^−2^	+ + +	1	+ + +	1	+ + +
10^−2^	2.2 × 10^−4^	+ + +	10^−2^	+ + (+)	10^−2^	+ + +
10^−4^	2.2 × 10^−6^	(− − −)	10^−4^	− − −	10^−4^	+ − −
10^−6^	2.2 × 10^−8^	− − −	10^−6^	− − −	10^−6^	− − −

**Table 5:** Selected mapping results in different samples containing a Tobamovirus, Begomovirus or viroid.

Targeted Taxon	Sample	Species (Accession)	MRD	Coverage (%)	CCB (%)
Tobamovirus	19/70	tomato mosaic virus (MH393623.1)	20,316	100	100
		ToBRFV (QBANK23289)	631	50.6	9.56
	23/1341	tobacco mild green mosaic virus (PP198314.1)	14,587	100	100
		ToBRFV (QBANK23289)	0	0	0
Begomovirus	EL 20 30031	tomato leaf curl Mali virus (MH794634.1)	7	85.9	53.4
		tomato geminivirus Senegal (AF058029.1)	5	94.6	59.9
		ToLCNDV DNA-A (QBANK22939)	0	0	0
		ToLCNDV DNA-B (QBANK22940)	0.5	18.6	11.5
	El 25 12/49	tomato leaf curl Comoros virus (NC_028114.1)	78	100	100
		ToLCNDV DNA-A (QBANK24440)	1.1	8.7	4.3
		ToLCNDV DNA-B (QBANK24441)	0	0	0
	17/104,1	chili leaf curl virus (LN845957.1)	316	100	100
		pepper leaf curl Bangladesh virus (DQ116881.1)	224	85	63.2
		croton yellow vein mosaic virus (FN645915.1)	102	68.6	54.3
		ToLCNDV DNA-A (QBANK24440)	1	8.7	8.7
		ToLCNDV DNA-B (QBANK22940)	0.11	7.19	7.19
viroid	08/01,1i17	tomato chlorotic dwarf viroid (FJ822877.1)	8359	100	100
		PCFVd (QBANK22265)	0	0	0
	16/324,3	dahlia latent viroid (JX263426.1)	978	99.7	99.7
		PCFVd (QBANK22265))	0	0	0

## Data Availability

The ENA accession numbers of the sequences reported in this paper are. Study: PRJEB113325. Samples: ERS30333753; ERS30333754; ERS30333755; ERS30333756; ERS30333757; ERS30333758; ERS30333759; ERS30333760; ERS30333761; ERS30333762; ERS30333763; ERS30333764; ERS30333765; ERS30333766; ERS30333767; ERA36324601.

## Supplementary Materials

The following supporting information can be downloaded at: https://www.mdpi.com/article/10.3390/v18060659/s1, Table S1: List of keywords used to filter the list of suspected viruses; Table S2: Run information and mapping results on PCFVd, ToBRFV and ToLCNDV for all samples; Table S3: Assignment results (limited to the nodes identified as viral by virhunter and assigned to sequences identified as viral in NCBI and to the nodes assigned to sequences identified as viroids); Table S4: Mapping results and confirmation (In grey, mapping results with coverage and CCB above the 33% threshold (virus, viroid or close relative considered present)).

## References

[1] Savary S., Willocquet L., Pethybridge S.J., Esker P., McRoberts N., Nelson A. (2019). The Global Burden of Pathogens and Pests on Major Food Crops. Nat. Ecol. Evol..

[2] Maclot F., Candresse T., Filloux D., Malmstrom C.M., Roumagnac P., van der Vlugt R., Massart S. (2020). Illuminating an Ecological Blackbox: Using High Throughput Sequencing to Characterize the Plant Virome Across Scales. Front. Microbiol..

[3] Lebas B., Adams I., Al Rwahnih M., Baeyen S., Bilodeau G.J., Blouin A.G., Boonham N., Candresse T., Chandelier A., De Jonghe K. (2022). Facilitating the Adoption of High-Throughput Sequencing Technologies as a Plant Pest Diagnostic Test in Laboratories: A Step-by-Step Description. EPPO Bull..

[4] (2022). PM 7/151 (1) Considerations for the Use of High Throughput Sequencing in Plant Health Diagnostics. EPPO Bull..

[5] Massart S., Adams I., Al Rwahnih M., Baeyen S., Bilodeau G.J., Blouin A.G., Boonham N., Candresse T., Chandellier A., De Jonghe K. (2022). Guidelines for the reliable use of high throughput sequencing technologies to detect plant pathogens and pests. Peer Community J..

[6] Massart S., Olmos A., Jijakli H., Candresse T. (2014). Current Impact and Future Directions of High Throughput Sequencing in Plant Virus Diagnostics. Virus Res..

[7] Maree H.J., Fox A., Al Rwahnih M., Boonham N., Candresse T. (2018). Application of HTS for Routine Plant Virus Diagnostics: State of the Art and Challenges. Front. Plant Sci..

[8] Sukhorukov G., Khalili M., Gascuel O., Candresse T., Marais-Colombel A., Nikolski M. (2022). VirHunter: A Deep Learning-Based Method for Detection of Novel RNA Viruses in Plant Sequencing Data. Front. Bioinform..

[9] Ren J., Ahlgren N.A., Lu Y.Y., Fuhrman J.A., Sun F. (2017). VirFinder: A Novel k-Mer Based Tool for Identifying Viral Sequences from Assembled Metagenomic Data. Microbiome.

[10] European and Mediterranean Plant Protection Organization (2021). PM 7/98 (5) Specific Requirements for Laboratories Preparing Accreditation for a Plant Pest Diagnostic Activity. EPPO Bull..

[11] Rong W., Rollin J., Hanafi M., Roux N., Massart S. (2023). Validation of High-Throughput Sequencing as Virus Indexing Test for *Musa* Germplasm: Performance Criteria Evaluation and Contamination Monitoring Using an Alien Control. PhytoFrontiers^TM^.

[12] Bester R., Cook G., Breytenbach J.H.J., Steyn C., De Bruyn R., Maree H.J. (2021). Towards the Validation of High-Throughput Sequencing (HTS) for Routine Plant Virus Diagnostics: Measurement of Variation Linked to HTS Detection of Citrus Viruses and Viroids. Virol. J..

[13] European and Mediterranean Plant Protection Organization (2024). Addendum—New Supporting Information for PM 7/151 Considerations for the Use of High Throughput Sequencing in Plant Health Diagnostics. EPPO Bull..

[14] Soltani N., Stevens K.A., Klaassen V., Hwang M.-S., Golino D.A., Al Rwahnih M. (2021). Quality Assessment and Validation of High-Throughput Sequencing for Grapevine Virus Diagnostics. Viruses.

[15] Pecman A., Kutnjak D., Gutiérrez-Aguirre I., Adams I., Fox A., Boonham N., Ravnikar M. (2017). Next Generation Sequencing for Detection and Discovery of Plant Viruses and Viroids: Comparison of Two Approaches. Front. Microbiol..

[16] Remenant B., Tian S., Bahut M., Porcher L., Rolland M. (2023). EVIDanses: A Quality Management-Friendly Bioinformatics Pipeline for Virus Detection in Plants. Proceedings of the Book of Abstracts.

[17] Buchfink B., Xie C., Huson D.H. (2015). Fast and Sensitive Protein Alignment Using DIAMOND. Nat. Methods.

[18] Jordan R., Guaragna M.A., Lockhart B., Oplaat C., Botermans M., de Koning P., Meekes E., Mollov D. (2024). Complete Genome Sequence of Shamrock Chlorotic Ringspot Virus, a Novel Potyvirus Infecting Ornamental Oxalis Triangularis in the United States and the Netherlands. Proceedings of the Acta Horticulturae.

[19] Chen S. (2023). Ultrafast One-Pass FASTQ Data Preprocessing, Quality Control, and Deduplication Using Fastp. iMeta.

[20] Andrey P., Dmitry A., Dmitry M., Alla L., Anton K. (2020). Using SPAdes De Novo Assembler. Current Protocols in Bioinformatics.

[21] Altschul S.F., Gish W., Miller W., Myers E.W., Lipman D.J. (1990). Basic Local Alignment Search Tool. J. Mol. Biol..

[22] Langmead B., Salzberg S.L. (2012). Fast Gapped-Read Alignment with Bowtie 2. Nat. Methods.

[23] Danecek P., Bonfield J.K., Liddle J., Marshall J., Ohan V., Pollard M.O., Whitwham A., Keane T., McCarthy S.A., Davies R.M. (2021). Twelve Years of SAMtools and BCFtools. GigaScience.

[24] Saison A., Gentit P. (2015). Development of a Polyvalent Detection Method for Begomoviruses Presenting a Threat to the European Tomato Industry.

[25] Accotto G.P., Navas-Castillo J., Noris E., Moriones E., Louro D. (2000). Typing of Tomato Yellow Leaf Curl Viruses in Europe. Eur. J. Plant Pathol..

[26] Kumar R., Palicherla S.R., Mandal B., Kadiri S. (2018). PCR Based Detection of Betasatellite Associated with the Begomoviruses Using Improved Universal Primers. Australas. Plant Pathol..

[27] Pappu H.R., Druffel K.L. (2009). Use of Conserved Genomic Regions and Degenerate Primers in a PCR-Based Assay for the Detection of Members of the Genus *Caulimovirus*. J. Virol. Methods.

[28] Untiveros M., Perez-Egusquiza Z., Clover G. (2010). PCR Assays for the Detection of Members of the Genus Ilarvirus and Family Bromoviridae. J. Virol. Methods.

[29] Lotos L., Efthimiou K., Maliogka V.I., Katis N.I. (2014). Generic Detection of Poleroviruses Using an RT-PCR Assay Targeting the RdRp Coding Sequence. J. Virol. Methods.

[30] Verhoeven J.T.J., Jansen C.C.C., Willemen T.M., Kox L.F.F., Owens R.A., Roenhorst J.W. (2004). Natural Infections of Tomato by Citrus Exocortis Viroid, Columnea Latent Viroid, Potato Spindle Tuber Viroid and Tomato Chlorotic Dwarf Viroid. Eur. J. Plant Pathol..

[31] Ha C., Coombs S., Revill P.A., Harding R.M., Vu M., Dale J.L. (2008). Design and Application of Two Novel Degenerate Primer Pairs for the Detection and Complete Genomic Characterization of Potyviruses. Arch. Virol..

[32] Sabanadzovic S., Valverde R.A., Brown J.K., Martin R.R., Tzanetakis I.E. (2009). Southern Tomato Virus: The Link between the Families *Totiviridae* and *Partitiviridae*. Virus Res..

[33] Li Y., Tan G., Lan P., Zhang A., Liu Y., Li R., Li F. (2018). Detection of Tobamoviruses by RT-PCR Using a Novel Pair of Degenerate Primers. J. Virol. Methods.

[34] Menzel W., Winter S. (2021). Identification of Novel and Known Tobamoviruses in Tomato and Other Solanaceous Crops Using a New Pair of Generic Primers and Development of a Specific RT-QPCR for ToBRFV. Acta Hortic..

[35] Villamor D.E.V., Ho T., Al Rwahnih M., Martin R.R., Tzanetakis I.E. (2019). High Throughput Sequencing For Plant Virus Detection and Discovery. Phytopathology.

[36] Adams I., Fox A., Wang A., Zhou X. (2016). Diagnosis of Plant Viruses Using Next-Generation Sequencing and Metagenomic Analysis. Current Research Topics in Plant Virology.

[37] Iandolino A.B., Goes da Silva F., Lim H., Choi H., Williams L.E., Cook D.R. (2004). High-Quality RNA, CDNA, and Derived EST Libraries from Grapevine (*Vitis Vinifera* L.). Plant Mol. Biol. Rep..

[38] Xu J., Aileni M., Abbagani S., Zhang P. (2010). A Reliable and Efficient Method for Total Rna Isolation from Various Members of Spurge Family (Euphorbiaceae). Phytochem. Anal..

[39] Lambert C., Braxton C., Charlebois R.L., Deyati A., Duncan P., La Neve F., Malicki H.D., Ribrioux S., Rozelle D.K., Michaels B. (2018). Considerations for Optimization of High-Throughput Sequencing Bioinformatics Pipelines for Virus Detection. Viruses.

[40] Rolland M., Villemot J., Marais A., Theil S., Faure C., Cadot V., Valade R., Vitry C., Rabenstein F., Candresse T. (2017). Classical and next Generation Sequencing Approaches Unravel Bymovirus Diversity in Barley Crops in France. PLoS ONE.

[41] Hagen C., Frizzi A., Gabriels S., Huang M., Salati R., Gabor B., Huang S. (2012). Accurate and Sensitive Diagnosis of Geminiviruses through Enrichment, High-Throughput Sequencing and Automated Sequence Identification. Arch. Virol..

